# Selection of Mx gene genotype as genetic marker for Avian Influenza resistance in Indonesian native chicken

**DOI:** 10.1186/1753-6561-5-S4-S37

**Published:** 2011-06-03

**Authors:** Tike Sartika, Sri Sulandari, Moch Syamsul Arifin Zein

**Affiliations:** 1 Indonesian Research Institute for Animal Production (IRIAP), PO BOX 221- Bogor 16002, Indonesia; 2 Research Centre for Biology, The Indonesian Institute of Sciences (LIPI), Cibinong 16911, Indonesia

## Abstract

**Background:**

In previous studies, the Mx Gene has been demonstrated to confer positive anti viral responses in chicken. The amino acid variation of Asn (allele A) at position 631 was specific to positive antiviral Mx/resistant, while, that of Ser (allele G) was specific to negative Mx/susceptible. This research was aimed at selecting one of the native chicken breeds which was found out to be resistant to avian influenza using molecular technique. The selected breed will then be used as the base population to improve native chicken breed in Indonesia.

**Methods:**

Marker Assisted Selection (MAS) method was used in this research to accelerate the selection process, since the disease resistance had low heritability value. Polymerase Chain Reaction-Restriction Fragment Length Polymorphism (PCR-RFLP) technique used to select the genotype of Mx^++^, Mx^+-^ and Mx^--^ that corresponded to the positive antiviral activity (Mx^++^), or those which had positive or negative activity (Mx^+-^) and negative antiviral activity (Mx^--^). There were 200 native hens and 40 cocks used in this experiment. Allele frequency of Mx Gene was calculated. The productivity indicators such as age at first laying, egg weight and hen weight at first laying and egg production were also measured. The chicken that had Mx^++^ and Mx^+-^ genotypes, were selected to produce offspring.

**Results:**

Result showed that the frequency of the resistant allele (Mx^+^) was 65% and 60% in laying hens and in cocks, respectively, while the frequency of the susceptible allele (Mx^-^) was 35% and 40% in hens and cocks, resepctively. Age, egg weight and hen weight at first laying and egg production for susceptible genotype were slightly better than for the resistant genotype which were 172,41 VS 178,81 days; 33,94 VS 32,84 g; 1450 VS 1439 g and 54,32 VS 48,30 %, respectively.

## Background

Indonesia has many varieties of native chicken. Based on phenotypic performances there are more than 32 distinctive breeds that are being raised under extensive and/or intensive systems [1]. Estimated population was about 230 millions. In Indonesia, meat from native chicken is more expensive than from commercial hybrid chicken. The consumers like to pay more as it is tastier and low fat content. The native chicken eggs are also more expensive than commercial chicken eggs, because it can be used as part of traditional herbal drink call “Jamu”, which is very popular in Indonesia.

Since 2003, Indonesia has outbreaks of Avian Influenza (AI). Naturally, native chicken has ability to resist the virus controlled by antiviral genes. The Mx proteins are key components and its coding protein had been shown to be induced by interferon (IFN) and to inhibit the replication of RNA virus [2]. Their genetic resistance was shown to result from the difference in genomic structure of the Mx gene. Watanabe [3] studied chicken Mx cDNAs from other breeds to see whether these chickens carried resistant or sensitive character of the Mx gene to the VSV/*vesicular stomatities virus* infection, as compared with the differential antiviral activity with amino acid substitutions at 15 positions. Only an amino acid substitution at position 631 was identified to determine the difference between the antiviral activity of chicken Mx protein; Asparagine (Asn) corresponded to the positively antiviral activity and serine (ser) corresponded to the negatively antiviral activity [2-4]. The chicken Mx protein spans about 2,118 bp, with 13 exons on chromosome 1 of the chicken genome. A total of 237 single nucleotide polymorphisms were found in the chicken Mx gene by comparison among 4 directly sequenced Mx genomic DNA sequences. In this study, identification of Mx gene by mismatching PCR-RFLP method can discriminate whether the chicken carry positive or negative virus activity. Sulandari *et al*[5] reported the study of 485 samples from 15 breeds of Indonesian native chicken by a specific PCR-RFLP technique showed that the averaged frequency of resistant allele (A/Mx^+^ allele) was 62.73% and that of sensitive allele (G/Mx^-^ allele) was 37.27%. Investigation of distribution of the allele A (Mx^+^) and G (Mx^-^) on chickens has been also reported [2,3,6-9].

The aim of the research was to examine the proportion of allele frequency of Indonesian Native chicken, especially Kampung chicken at breeding population in IRIAP (*Indonesian Research Institute for Animal Production*). Selection using resistant Mx gene is effective for breeding program to increase selected breed as resistant to RNA virus.

## Methods

A total of 240 samples (200 hens and 40 cocks) from one of the native chickens in Indonesia (Kampung chicken, selected for egg production for 6 generations) were used in this study. The fresh blood from chickens was collected and preserved in 96% absolute alcohol. Genomic DNA was extracted from whole blood using the phenol-chloroform method [10]. PCR-RFLP method was used to genotype the G/A SNP at nucleotide position 1,892 in the 13^th^ exon of coding sequence of the Mx gene using PCR-RFLP mismatched primers. The mismatch primer sequences [8] which amplify approximate 100 bp long fragment were as follows: Forward primer NE-F2 (5’CCTTCAGCCTGTTTTTCTCCTTTTAGGAA3’) and Reverse primer NE-R2/R (5’CAGAGGAATCTGATTGCTCAGGCGTGTA3’) or Reverse primer NE-R2/S (5’CAGAGGAATCTGATTGCTCAGGCGAATA3’). The Rsa1 restriction enzyme was used with a recognition sequence of 5’GT↓AC3’ to cut the fragment at the position of interest when there is an allele G using primer NE-F2 and NE-R2/R, while the Ssp1 restriction enzyme was used with a recognition sequence of 5’AAT↓ATT3’ to cut the fragment at the position of an allele A using primer NE-F2 and NE-R2/S.	The following PCR condition was used: an initial denature at 94°C for 5 min, followed by 35 cycles of 60 s at 94°C, annealing temperature for 60 s at 60°C, and 72°C for 60 s, and final extension at 72°C for 5 min. PCR product were analyzed by electrophoresis through 2% agarose gel in 1x TAE buffer, and stained with ethidium bromide. Amplicons were cleaved with the restriction enzyme the Rsa1 and/or Ssp 1 (1U/µg) for 6-8 hours at 37°C following the manufacture’s instruction. The digested fragments were visualized by 12% polyacrylamide gel in constant voltage 160 volt for 4 hours. The gel was stained with silver nitrate [11] and scanned using Adobe Photoshop.

Allele frequencies were calculated for hens and cocks. Productivity such as age, egg weight and hen weight at first laying and egg production based on genotype Mx gene were analysed using Anova, Minitab V.14.

## Results and discussion

### Mx gene genotyping

**Figure 1:**
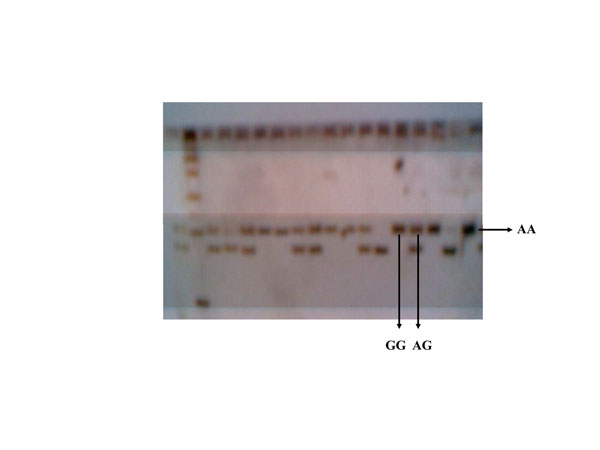
**Genotyping of Mx gene by acrylamide gel** AA, Genotype of Mx gene: resistant/resistant AG, Genotype of Mx gene: resistant/sensitive GG, Genotype of Mx gene: sensitive/sensitive

The genomic DNA of 200 hens and 40 cocks were successfully amplified. Identification of resistant and sensitive chicken Mx gene was examined by mismatch PCR-RFLP. The PCR product was cleaved with the restriction enzyme of the Rsa1 and the digested showing polymorphism bands, one band with 100 bp in length (A/A, homozygous resistant Mx allelic genes); two bands with 100 bp and 73 bp in length (A/G, heterozygous Mx allelic genes); and one band with 73 bp in length (G/G, homozygous sensitive Mx allelic gene). An example of genotyping results is presented in Figure 1, and corresponding allele frequency is presented in Table 1.

**Table 1:** Frequency of allelic hen and cock native chicken

Samples	Genotype			Frequency of allele
	AA/Mx^++^	AG/Mx^+-^	GG/Mx^--^	
Hens native chicken (200)	80	102	18	f(A) =0,65 f(G) =0,35
Cocks native chicken (40)	15	18	7	f(A) = 0,60 f(G) = 0,40

As shown in Table 1, results from the 240 samples by specific PCR-RFLP indicate a polymorphism at the Mx gene (which is putatively associated with AI resistance/susceptibility in chicken). The frequency of the resistance allele (A allele) for the hen native chicken was 65%, while for cock native chicken was 60%, and that for sensitive allele (G allele) was 35% for hen and 40% for cock native chickens. Investigation of the distribution of the allele A and G on chickens has also been reported [2,3,5-8,12]. As reported by Sulandari *et al*., [5],Indonesian native chicken had averaged frequency of resistant allele (A allele) of 62.73% and sensitive allele (G allele) of 37.27%. A representative various breed of Indonesian native chicken such as White Kedu, Golden Arab, Sentul, Dwarf, Black Kedu, Pelung, Gaok, Kalosi, Tolaki, Merawang, and Cemani chickens, tend to have a higher frequency of the resistant allele. Frequency of A allele in each breed was 0.58, 0.62, 0.63, 0.66, 0.68, 0.69, 0.70, 0.70, 0.74, 0.81, and 0.87 respectively, while Kapas, Wareng, Nunukan and Silver Arab chickens, as founder local chicken, had a higher frequency of the sensitive allele, with a frequency of A allele of 0.32, 0.44, 0.45, and 0.47 respectively.

### Productivity of native chicken

The productivity of native chicken was divided by 3 groups for the Mx genotype presented (Table 2). Statistical analyses showed that for all traits no significant effect of the genotype. However, descriptively the GG genotype birds carrying the sensitive allele were relatively slightly better than those carrying the AG/resistant and sensitive allele and AA genotype/resistant allele. Age at first laying in GG genotype was lower than the AG and AA genotype. Similarly, egg weight, hen weight at first laying and egg production was better in GG genotype.

**Table 2:** The productivity of hen native chicken for 10 weeks at 1st periods lay

	Genotype		
	AA/Mx^++^	AG/Mx^+-^	GG/Mx^--^

Age at first laying Means (days) Sdv (days) CV (%) Max (days) Min (days)	178,81 16,15 9,03 221 149	174,28 13,79 7,91 216 152	172,41 15,20 6,97 202 155

Egg weight at first laying Means (g) Sdv (g) CV (%) Max (g) Min (g)	32,84 3,54 10,77 46 26	32,85 3,88 11,82 49 26	33,94 4,10 1,88 42 29

Hen weight at first laying Means (g) Sdv (g) CV (%) Max (g) Min (g)	1439,11 229,31 15,93 2437 962	1395,26 189,27 13,56 2081 1044	1450,06 231,94 106,39 2023 1197

Egg Production during 10 weeks Means (eggs) Sdv (eggs) CV (%) Max (eggs) Min (eggs) Means (%) Sdv (%) CV (%) Max (%) Min (%)	37,20 15,11 40,62 69 9 48,30 19,62 40,62 89,61 11,69	39,42 13,75 34,88 69 3 51,19 17,86 34,88 89,61 3,90	41,82 16,36 7,51 66 2 54,32 21,25 9,75 85,71 2,60

**Figure 2:**
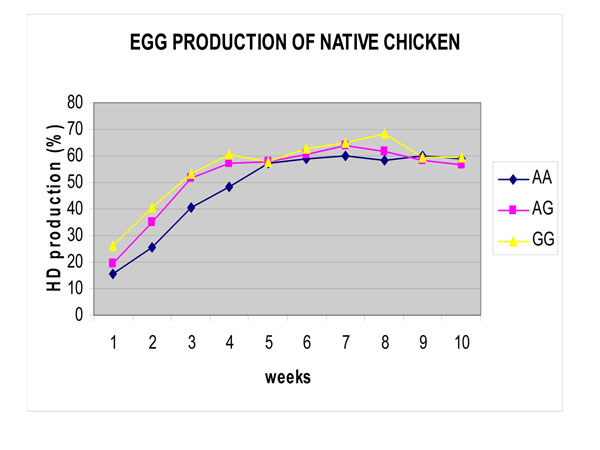
Egg production of native chicken

Hen day production curve was presented in Figure 2. Based on genotype Mx gene, hen day peak production of hen with GG genotype (64%) was slightly better than of hen with AA and AG genotype (60%). Egg production of native chicken in this study was better than in common native chicken (28%) in Indonesia [13]. Overall, the results indicated that the sensitive allele/ GG genotype tended to be better in productivity than the resistant allele/AA genotype.

## Conclusion

In conclusion, the frequency of Mx^+^ gene (A allele) in Indonesian native chicken was relatively high. The productivity of native chicken measured as age, egg weight, body weight at first laying and 12 weeks egg production didn’t differ significantly between Mx genotypes.

## Competing interests

The author(s) declare that they have no competing interests.
